# A PRACTICE FRAMEWORK FOR CONSIDERATION OF GENDER IN ETHICAL DECISION-MAKING

**DOI:** 10.1093/geroni/igz038.889

**Published:** 2019-11-08

**Authors:** Sharon Bowland, Beth Halaas

**Affiliations:** 1 University of North Texas, Denton, Texas, United States; 2 Eastern Washington University, Cheney, Washington, United States

## Abstract

Gender role stereotypes, social norms and social policies negatively influence health and well-being for marginalized groups. These inequalities are embedded in the fabric of our society and are often unquestioned and hidden. Practitioners frequently use an ethical lens that does not consider the influence of gender on life course decision-making. We developed the Practice Framework for Older Persons (PFOP) to assess past and current realities that take gendered experiences into consideration. By contextualizing means and opportunities, a more complete picture can be drawn about a person’s unique gender experiences. Subsequently, we can better understand their decision-making processes, wants, needs, and desires. This type of assessment may be particularly beneficial for women and transgender persons given ethical demands for practice paradigms which consider gender fluidity and development of a sense of personal agency.

